# The impact of a *Saccharomyces cerevisiae* bio-protective strain during cold static clarification on Catarratto wine

**DOI:** 10.3934/microbiol.2025003

**Published:** 2025-01-09

**Authors:** Enrico Viola, Vincenzo Naselli, Rosario Prestianni, Antonino Pirrone, Antonella Porrello, Filippo Amato, Riccardo Savastano, Antonella Maggio, Micaela Carusi, Venera Seminerio, Valentina Craparo, Azzurra Vella, Davide Alongi, Luca Settanni, Giuseppe Notarbartolo, Nicola Francesca, Antonio Alfonzo

**Affiliations:** 1 Department of Agricultural, Food and Forest Sciences (SAAF), University of Palermo, Viale delle Scienze, Bldg. 5, 90128, Palermo, Italy; 2 Department of Biological, Chemical and Pharmaceutical Sciences and Technologies (STEBICEF), University of Palermo, Viale delle Scienze, Bldg. 16, 90128, Palermo, Italy; 3 Az. Agr. G. Milazzo - Terre Della Baronia S.r.l., S.S. 123 km. 12+70, 92023, Campobello di Licata, Italy

**Keywords:** bio protection, *Saccharomyces cerevisiae*, sulphite-free, dissolved O_2_, sensory attributes, absorbance

## Abstract

The study aimed to evaluate the impact of the early addition of a *Saccharomyces cerevisiae* HD A54 strain before pressing during winemaking. This approach aimed to reduce the dissolved oxygen in the grape must, thus preserving the wine characteristics. Two different treatments were settled: Trial A, where sulphite or other substances were not added during pressing; and Trial B, where a *S. cerevisiae* strain was added at the pressing stage. The chemical parameters were determined through an enzymatic analyzer, which indicated a faster fructose consumption compared to the glucose in Trial A. The plate counts were measured to monitor the microbial groups during vinification. Both treatments showed regular trends with respect to the *Saccharomyces* population. Trial B exhibited a higher oxygen consumption compared to the control trial, especially in the early stages of winemaking. This was determined through a dissolved O_2_ analysis. Furthermore, Trial B had lower absorbance values at the post-pressing and pre-clarification stages. Both the dissolved oxygen and the absorbance analyses underscored the positive impact of the *S. cerevisiae* HD A54 strain in protecting against oxidative processes in the grape musts at the pre-fermentative stage. The analysis of volatile organic compounds detected 30 different compounds, including alcohols and esters. Trial B had higher alcohol levels, particularly hydroxyethylbenzene (135.31 mg/L vs. 44.23 mg/L in Trial A). Trial A had almost a four times higher ethyl acetate concentration than Trial B, which is an indicator of oxidation. Interestingly, Trial B showed higher concentrations of 3-methyl-butyl acetate and 2-phenylethyl acetate, which are molecules that correspond to fruity (banana) and floreal (rose) aromas, respectively. Regarding the sensory analysis, Trial B received better scores for the fruity and floral attributes, as well as the overall wine quality.

## 1. Introduction

Over the past few decades, the winemaking industry has placed a significant reliance on sulphur dioxide (SO_2_) as a preservative due to the numerous critical functions it performs [1]. In the context of winemaking, SO_2_ is an effective agent to combat acetic acid bacteria, lactic acid bacteria, and yeasts, thus maintaining the quality of the wine. Additionally, it acts as a potent antioxidant, thereby mitigating the effects of dissolved oxygen and inhibiting oxidising enzymes. Grape oxidases (tyrosinase and laccase), which are responsible for phenol oxidation and aroma development, are neutralised by SO_2_ [2]. This compound is a broad-spectrum antimicrobial agent that prevents off-flavor formation by inhibiting the indigenous microbiota that could otherwise lead to uncontrolled spontaneous fermentation [3]. SO_2_ enhances the wine color stability during ageing and facilitates the release of phenolic compounds from the grape skin during maceration [4]. Due to its affordability and ability to maintain wine characteristics even after bottling, SO_2_ is widely used in winemaking globally [5]–[8]. However, there are concerns about excessive SO_2_ levels. The World Health Organization (WHO) has recommended the use of alternative methods to either reduce or eliminate the consumption of sulphites, particularly in light of the potential negative effects on consumer health, especially for those with allergies or sensitivities [2]. Additionally, the International Organization of Vine and Wine (OIV) has been gradually lowering the maximum recommended dosage for distributed wines [9]. Furthermore, the European Community has mandated that wine containing SO_2_ must include this information on labels [10]. Moreover, global wine consumers have demonstrated a growing inclination towards products that align with the natural definition, which is characterized by a minimal use of preservatives and chemicals. The objective is to espouse ecologically responsible wine-growing and oenological practices that prioritize consumer health [11],[12].

In order to control the microbial populations in the context of winemaking, a number of alternative strategies have been explored, including the use of ultraviolet radiation, pulsed electric fields (PEF), high pressure, ultrasound, and high hydrostatic pressure treatments [13],[14]. Furthermore, researchers have investigated the potential of utilizing substances such as chitosan, lysozyme, dimethyl dicarbonate, and sorbic acid to address this challenge [6]. In addition to the aforementioned physical and chemical methods, there is a biological control alternative, known as bio-protection. The strategy is comprised of a series of techniques designed to prevent microbial contamination and to achieve the same results typically achieved through the use of SO_2_. In particular, various microbial species, including yeasts (both *Saccharomyces* and non-*Saccharomyces*) and bacteria, are introduced at different stages of the food production process, either before, during, or after the process [15]. These microorganisms act as bio-protectors by employing a range of mechanisms, comprising both passive and active strategies. Passive strategies include the deprivation of resources and the establishment of dominance within a colonized space [16]–[18]. Microorganisms deploy tactics to restrict the competitors' access to resources, including nutrients and oxygen. Furthermore, they secure dominance in terms of the space they occupy. Indeed, the nutrients, oxygen, and space are the main parameters that affect the population dynamics [19]–[22]. Conversely, active strategies include the production of several molecules with various effects, such as antimicrobial compounds [15]. Notably, to date, no studies have evaluated the effect of inoculating a low concentration of *Saccharomyces cerevisiae* before the pressing stage to enhance the defense against oxygen-related issues.

## 2. Materials and methods

### 2.1. Experimental design and winemaking process

**Figure 1.**
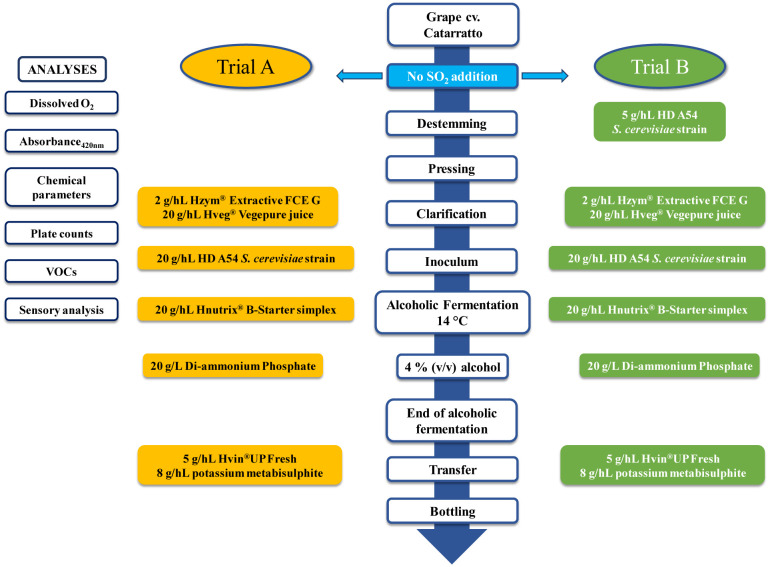
Experimental design of Catarratto wine vinified with or without *Saccharomyces cerevisiae* bio-protective HD A54 strain.

The experimental plan is reported in Figure 1, and foresaw to evaluate the effect on oxygen consumption, organoleptic properties, and volatile compounds using *Saccharomyces cerevisiae* strains during the white vinification process. The Catarratto variety (*Vitis vinifera* L.) of grapes from a vineyard located in San Giuseppe Jato, (37°59′20′′ N; 13°11′34′′ E, Palermo, Sicily, Italy) were processed at a laboratory scale in the Department of Agricultural, Food and Forest Sciences (SAAF) at University of Palermo. Immediately after harvesting, the grapes were destemmed, crushed, and pressed without the addition of any coadjuvant (Trial A). In the second trial (B), 5 g/hL of the *S. cerevisiae* HD A54 strain was added at the pressing stage. The strain was used in its active dry yeast (ADY) form, and was rehydrated following the manufacturer's protocol. Clarification was carried out and lasted 24 h at 8 °C. The following products were used for clarification: Clarification Hzym® Extractive FCE G (2 g/hL; HTS Enologia, Marsala, Italy) and Hveg® Vegepure juice (20 g/hL; HTS Enologia, Marsala, Italy). SO_2_ was not added in “A” trial, nor in trial “B” before clarification. The *Saccharomyces cerevisiae* HD A54 strain, provided by HTS Enologia (Marsala, Italy), was used as a starter to carry out alcoholic fermentation, which was carried out at 14 °C. Prior to yeast inoculum, each trial was supplemented with 20 g/hL of Hnutrix® B-Starter simplex (HTS Enologia, Marsala, Italy), which is a yeast partial autolisate useful as a starting nutrient for ADY. Di-ammonium phosphate (DAP; 20 g/L; Chimica Noto s.r.l., Partinico, Italy) was added during alcoholic fermentation at 4% (v/v) of ethanol; during the last phase of alcoholic fermentation, 10 g/hL of Hnutrix® B-Energia (HTS Enologia, Marsala, Italy), which is a formulation of organic nitrogen and micronutrients, was added in order to avoid nutrient starvation during alcoholic fermentation. At the end of alcoholic fermentation, the wines were separated from the lees and transferred into a clean glass carboy; lastly, 5 g/hL of Hvin®UP Fresh (HTS Enologia, Marsala, Italy), a solution used to improve shelf life and to preserve wine aroma, and 8 g/hL of potassium metabisulphite were added. The wines were stored at 8 °C up to the bottling phase. Sample collection involved the grape must during several phases of the vinification process. In detail, the grape must was collected after pressing, before, during (12 h of process ongoing), and after clarification, at yeast inoculum, at 3 and 6 days of alcoholic fermentation, and at the end of alcoholic fermentation. All samples were immediately subjected to analyses.

### 2.2. Microbiological analyses

All samples collected during the wine production were analyzed; in particular, the yeast and lactic acid bacteria (LAB) populations were monitored. The samples were diluted in Ringer's solution (Sigma-Aldrich, Milan, Italy) (ratio 1:10) and analyzed in triplicate for total yeasts (TY) on Wallerstein Laboratory (WL) nutrient agar (Condalab, Torrejón de Ardoz, Spain) [23] at 28 °C for 72 h, and total non-*Saccharomyces* yeast on Lysine Agar (LA) (Thermo Fisher Scientific Inc., Milan, Italy) at 28 °C for 5 days [24]. Mesophilic lactobacilli were detected on de-Man, Rogosa, and Sharpe (MRS) agar medium (Condalab, Torrejón de Ardoz, Spain), which were supplemented with cycloheximide (10 mg/mL) and incubated under microaerophilic conditions at 30 °C for 48 h [25]. Presumptive *Saccharomyces* spp. were calculated as the difference between the total yeast count on WL nutrient agar and the total non-*Saccharomyces*.

### 2.3. Physicochemical analyses

#### 2.3.1. Wine composition

Regarding the chemical parameters monitored during alcoholic fermentation, the pH and total acidity were assessed following the OIV procedures [26],[27]. In addition, the remaining monitored chemical parameters were detected through enzymatic assays on an iCubio iMagic M9 (iCubio Biomedical Technology Co. Ltd., Shenzhen, China); in detail, the quantification of acetic, malic, tartaric, and lactic acids was conducted as reported by Matraxia et al. [28]. Furthermore, the ethanol content was measured as previously reported by Chawafambira [29]. The reagents were purchased from R-Biopharm AG (Darmstadt, Germany), and these analyses were performed in triplicate.

#### 2.3.2. Oenological parameters

The soluble oxygen was monitored to evaluate the effect of the *S. cerevisiae* HD A54 strain to reduce the oxygen content from post pressing to the end of alcoholic fermentation. To this end, a portable dissolved oxygen meter Mod. HQ30D, equipped with Intellical LDO101 Luminescence/Optical dissolved oxygen probe (Hach Lange S.r.l, Milan, Italy), was employed. The samples were analyzed for the absorbance using a 420 nm optical path before, during, and after clarification, at the inoculum, and at the end of alcoholic fermentation by spectrophotometer (UV-1601-Shimadsu, Tokyo, Japan).

### 2.4. Analysis of Volatile Organic Compounds (VOCs) in wine samples

#### 2.4.1. Standard solutions

The standards for each compound were individually purchased from Sigma-Aldrich (82024 Taufkirchen, Germany). 2,3-butanediol was used as the standard for the alcohol fraction, acetoin as the standard for the carboxyl-function fraction, and ethyl lactate as the standard for the ester fraction. In addition, n-alkane standards (C8 to C40) were purchased from the Aldrich Chemical Co. (St. Louis, Mo., USA). The standard solutions of each compound were prepared at five different concentrations: 2,3-butanediol (53.2 mg/L, 112.5 mg/L, 225.0 mg/L, 262.0 mg/L, and 450.0 mg/L); acetoin (24.7 mg/L, 45.70 mg/L, 64.7 mg/L, 115.6 mg/L, 173.30 mg/L, and 289.8 mg/L); and ethyl lactate (79.0 mg/L, 134.0 mg/L, 224.0 mg/L, 326.0 mg/L, and 477.0 mg/L).

#### 2.4.2. Extraction, identification and quantification of VOCs by GC-MS

In order to determine the volatile organic composition, the procedure outlined by Francesca et al. [30] was applied. In detail, the wine samples (10 mL) from all trials were mixed with MS SupraSolv® dichloromethane (5 mL) in a 50 mL conical flask. The mixture was stirred at room temperature for 30 min and then centrifuged at 4000 rpm for 10 min using a Low-Speed Centrifuge (ScanSpeed 416) with a Swing Rotor (LaboGene ApS Industrivej 6–8, Vassingerød, DK-3540 Lynge, Denmark). The aqueous phase was removed, and anhydrous sodium sulphate (1 g) was added before centrifugation at 4000 rpm for 5 min. The dichloromethane layer was removed and dried under N_2_ gas to 0.3 mL.

Gas chromatographic analyses were performed with the Agilent 7000C GC system, fitted with a fused silica Agilent DB-5MS capillary column (30 m × 0.25 mm i.d.; 0.25 µm film thickness), coupled to an Agilent triple quadrupole Mass Selective Detector MSD 5973 (ionization voltage 70 eV; electron multiplier energy 2000 V; transfer line temperature 295 °C; solvent delay: 3.5 min). Helium was the carrier gas (1 mL/min).

The temperature was initially maintained at 40 °C for 1 min. Then, it was gradually increased to 250 °C at a rate of 3 °C/min for 30 min, and finally maintained at 250 °C at 10 °C/min. One µL of sample was automatically injected at 250 °C and in the splitless mode, where the transfer line temperature was 295 °C. The individual peaks were analyzed using the GC MS Solution package, Version 2.72. The identification of compounds was carried out using Adams, NIST 11, Wiley 9, and the FFNSC 2 mass spectral database. These identifications were also confirmed by other published mass spectra and linear retention indices (LRI). The LRIs were calculated using a series of n-alkanes (C8–C40). Quantification was carried out using the three calibration lines. For compounds belonging to other classes than the standards, similarity was used for quantification.

### 2.5. Quantitative descriptive sensory analysis

In order to determine the sensory profiles of the experimental wines, a quantitative descriptive analysis was conducted by following the methodology reported by Jackson [31].

Each panelist had a strong experience on winemaking and sensory analyses, and were picked among people who already participated in past similar studies as judges. There was a total of 14 trained judges, consisting in 7 men and 7 women between the ages of 26 and 63 years old. In order to assess their sensory skills, they were previously subjected to a test based on flavors and tastes associated to the wines. Several descriptive attributes were chosen to evaluate the experimental wines in terms of the odor appearance, mouth feel, gustatory taste, flavor, and overall quality. Other descriptors regarded the identification and quantification of off-odors and off-flavors connected to microbial aspects and a list of several other attributes referring to pungent, putrid, and petroleum [32].

Each individual attribute was assessed by scoring on an unstructured 9 cm scale. Lastly, every panelist judged each sample in triplicate with a different wine bottle every time. An incomplete balanced block design was utilized to reduce the contrast impact between the samples [33].

### 2.6. Statistical analysis

The XLStat software for Excel, version 2020.3.1 (Addinsoft, New York, USA), was used to process the statistical data and to generate the graphics. In particular, the physicochemical, microbiological, oenological, VOC, and sensory data were submitted to a one-way analysis of variance (ANOVA). The Tukey's test was performed to make the comparisons, and statistical significance was attributed to p < 0.05.

## 3. Results and discussion

### 3.1. Microbiological monitoring

**Figure 2.**
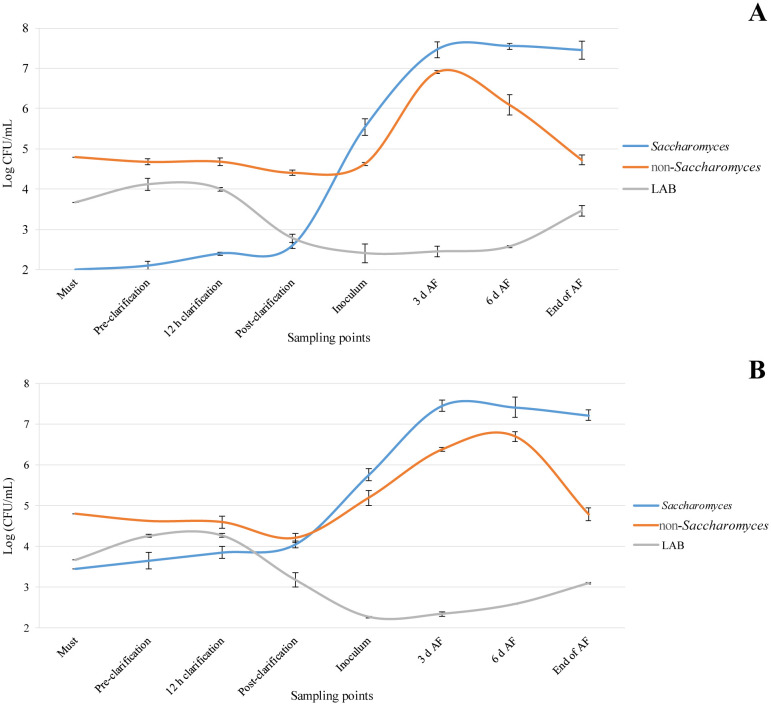
Trend of Yeast and bacteria population (Log CFU/mL) during alcoholic fermentation: (A) Trial A microorganism levels; (B) Trial B microorganism levels. Abbreviations: AF, alcoholic fermentation; LAB, lactic acid bacteria.

During fermentation, the yeast populations were investigated (Figure 2), and the indigenous non-*Saccharomyces* population in the grape must was found to be 4.8 Log CFU/mL. Notably, the *Saccharomyces* species was not detected in the grape must, which is consistent with previous studies [34]–[36]. Additionally, low concentrations of LAB were observed at the start of the vinification process (<4.0 Log CFU/mL). However, these LAB levels are comparable to those found by Sannino et al. [37] on SO_2_ free grape must. In Trial B, the *S. cerevisiae* HD A54 strain was inoculated during the pressing phase at a density between 3.4–3.5 Log CFU/mL. In Trial A, the presumptive *S. cerevisiae* population was slightly above the detection limit (2.1 Log CFU/mL). Interestingly, the presumptive *S. cerevisiae* levels remained stable during the clarification phase in Trial A, while there was a small increase in the presumptive *S. cerevisiae* levels in Trial B due to cell multiplication of the inoculated *S. cerevisiae* HD A54 strain. Although no literature studies specifically address the addition of bioprotective *S. cerevisiae*, this increase can be considered a regular growth pattern for an inoculated *S. cerevisiae* strain within a few hours [38].

In both Trials A and B, there was a consistent trend in non-*Saccharomyces* count values, with a slight decrease observed. The *S. cerevisiae* HD A54 strain was inoculated at a level of 6.0 Log CFU/mL in these trials. During alcoholic fermentation, both the presumptive *Saccharomyces* and presumptive non-*Saccharomyces* populations increased, reaching their peak at three days in Trial A and up to six days in Trial B. The fermentation kinetics of the *S. cerevisiae* population followed a regular pattern (Figure 2), which aligns with the findings of Morgan et al. [39] regarding SO_2_-free grape must.

### 3.2. Physicochemical analyses

#### 3.2.1. Wine composition

**Figure 3.**
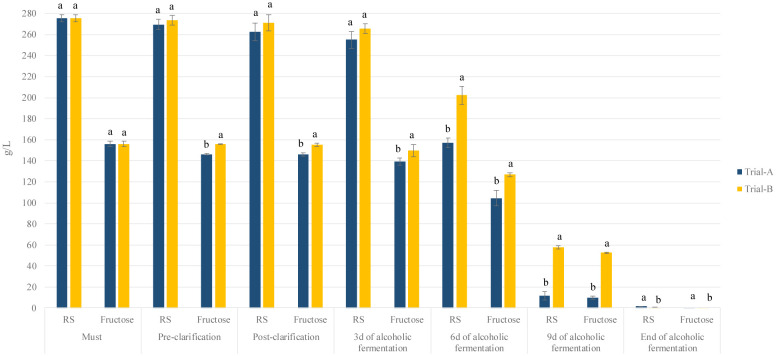
Bar chart of glucose and fructose detected throughout the vinification process. Result indicates mean value ± standard deviation of two determinations from three replicates. Data displaying different letters at the top of each bar are significantly different (P value < 0.05), according to Tukey's test. Abbreviations: RS, residual sugar.

The key chemical parameters (sugars, organic acids, pH, total acidity, and glycerol) determined in the must and wine are reported in Table 1. Initially, the musts showed a sugar concentration of 275.7 g/L, a pH of 3.25, tartaric acid at 5.08 g/L, and malic acid at 1.36 g/L. The sugar consumption and acetic acid production showed similar trends between the treatments during alcoholic fermentation. However, the fructose values significantly differed, with Trial A showing consumption due to indigenous non-*Saccharomyces* yeasts that favor fructose [40]. Despite this, both treatments reached similar residual sugar levels (Figure 3). At the end of fermentation, the acetic acid levels were comparable to those reported by Alfonzo et al. [41] on “Catarratto bianco lucido” cultivar, which was likely influenced by the bioprotective effect of the HD A54 strain. Indeed, several *S. cerevisiae* strains exert their antimicrobial effect through the production of killer toxins or other inhibitory substances [42]. Alcoholic fermentation concluded in 12 days for both treatments, which resulted in residual sugar values below 2.0 g/L.

Throughout the vinification process, no significant differences were found between the trials in terms of the acetic, malic, tartaric, or lactic acid, and the glycerol content. The ethanol levels were monitored at various sampling points during fermentation. Initially, both treatments showed similar ethanol values; however, by the sixth day, Trial A had a higher ethanol content (5.88 %) compared to Trial B (3.46 %). This trend continued, with Trial A reaching 13.34 % ethanol by the ninth day, while Trial B remained at 11.06 %. Ultimately, both treatments completed alcoholic fermentation with a similar ethanol content (14.10 % for Trial A and 14.04 % for Trial B), thus indicating successful fermentation.

**Table 1.** Chemical parameters determined during the winemaking process.

Parameters	Must	Vinification								
		Pre-clarification			Post-clarification			3 d of alcoholic fermentation		
		A	B	S.S.	A	B	S.S.	A	B	S.S.
Residual sugar Ψ	275.70 ± 3.41	269.6 ± 4.81a	273.80 ± 4.76a	n.s.	262.67 ± 8.44a	271.19 ± 7.74a	n.s.	255.05 ± 7.97a	265.61 ± 4.57a	n.s.
Fructose Ψ	156.15 ± 2.31	146.28 ± 0.46b	156.01 ± 0.46a	***	146.10 ± 1.55b	155.39 ± 1.59a	**	139.40 ± 3.51a	149.66 ± 6.04a	n.s.
Glicerol Ψ	0.00 ± 0.00	0.05 ± 0.01a	0.04 ± 0.01a	n.s.	0.115 ± 0.00a	0.11 ± 0.00a	n.s.	0.54 ± 0.02a	0.35 ± 0.13a	n.s.
Acetic acid Ψ	0.00 ± 0.00	0.00 ± 0.00a	0.00 ± 0.00a	n.s.	0.00 ± 0.00a	0.00 ± 0.00a	n.s.	0.08 ± 0.02a	0.07 ± 0.01a	n.s.
Malic acid Ψ	1.36 ± 0.03	1.37 ± 0.02a	1.36 ± 0.00a	n.s.	1.37 ± 0.03a	1.35 ± 0.00a	n.s.	1.29 ± 0.07a	1.31 ± 0.01a	n.s.
Tartaric acid Ψ	5.08 ± 0.05	4.33 ± 0.05a	4.56 ± 0.24a	n.s.	4.04 ± 0.19a	4.09 ± 0.00a	n.s.	4.85 ± 0.30a	4.42 ± 0.15a	n.s.
Lactic acid Ψ	0.00 ± 0.00	0.00 ± 0.00a	0.00 ± 0.00a	n.s.	0.00 ± 0.00a	0.00 ± 0.00a	n.s.	0.02 ± 0.01a	0.03 ± 0.00a	n.s.
Ethanol 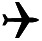	0.00 ± 0.00	0.22 ± 0.07a	0.09 ± 0.04a	n.s.	0.39 ± 0.16a	0.14 ± 0.09a	n.s.	0.84 ± 0.22a	0.41 ± 0.23a	n.s.
Parameters		6 d of alcoholic fermentation			9 d of alcoholic fermentation			End of alcoholic fermentation		
		A	B	S.S.	A	B	S.S.	A	B	S.S.
Residual sugar Ψ		157.00 ± 4.49b	202.25 ± 8.38a	***	11.75 ± 3.89b	57.60 ± 1.40a	***	1.53 ± 0.00a	0.51 ± 0.06b	***
Fructose Ψ		104.51 ± 7.48b	126.77 ± 2.08a	**	9.63 ± 1.36b	52.61 ± 0.52a	***	0.47 ± 0.06a	0.07 ± 0.07b	**
Glicerol Ψ		4.98 ± 0.16a	6.20 ± 1.62a	n.s.	6.58 ± 0.04a	7.12 ± 0.40a	n.s.	7.05 ± 0.37a	6.77 ± 0.25a	n.s.
Acetic acid Ψ		0.20 ± 0.03a	0.17 ± 0.01a	n.s.	0.10 ± 0.01a	0.10 ± 0.03a	n.s.	0.07 ± 0.01a	0.07 ± 0.03a	n.s.
Malic acid Ψ		1.25 ± 0.01a	1.24 ± 0.04a	n.s.	1.23 ± 0.05a	1.24 ± 0.10a	n.s.	1.21 ± 0.05a	1.20 ± 0.05a	n.s.
Tartaric acid Ψ		4.44 ± 0.11a	4.11 ± 0.13b	*	4.17 ± 0.17a	3.89 ± 0.17a	n.s.	3.55 ± 0.05a	3.67 ± 0.17a	n.s.
Lactic acid Ψ		0.01 ± 0.00a	0.02 ± 0.01a	n.s.	0.00 ± 0.00a	0.00 ± 0.00a	n.s.	0.00 ± 0.00a	0.00 ± 0.00a	n.s.
Ethanol 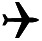		5.88 ± 0.25a	3.46 ± 0.31b	***	13.34 ± 0.21a	11.06 ± 0.12b	***	14.04 ± 0.03a	14.10 ± 0.04a	n.s.

Result indicates mean value ± standard deviation of two determinations from three replicates. Data within a row followed by the same letter are not signiﬁcantly different according to Tukey's test. Tukey's test was not applied on must values. P value: *, P < 0.05 **, P < 0.01; ***, P < 0.001. Ψ, expressed in g/L; 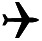, expressed as % (v/v). Abbreviations: SS, statistical significance; n.s., not significant.

#### 3.2.2. Oenological parameters

**Figure 4.**
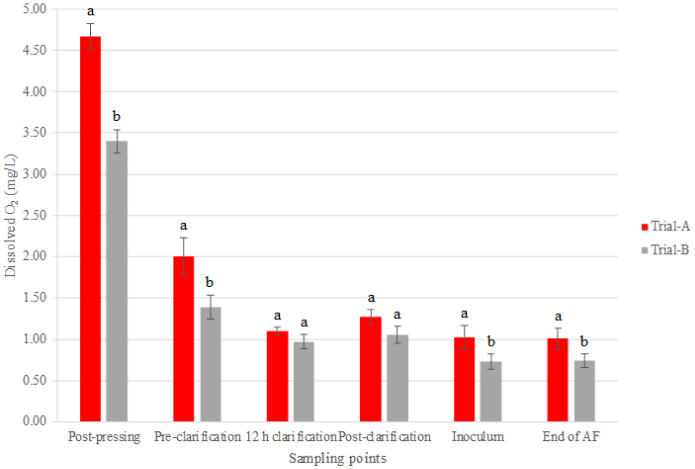
Bar chart of dissolved O_2_ detected in must during pre-fermentative phase. Result indicates mean value ± standard deviation of two determinations from three replicates. Data within a sampling point followed by the same letter are not signiﬁcantly different according to Tukey's test. Data followed by different letters are significantly different (P value < 0.05). Abbreviations: AF, alcoholic fermentation.

The monitoring of oxygen levels during the clarification process is depicted in Figure 4. Following the completion of the pressing process, the oxygen concentration was found to be 4.67 mg/L in Trial A. In comparison, Trial B, which included the bio-protective HD A54 strain, demonstrated a significantly lower oxygen concentration of 3.40 mg/L. As stated by Catarino et al. [43], several winemaking operations, particularly the pressing stage, contribute to the uptake of oxygen. The considerable difference in the dissolved oxygen levels between Trial A and Trial B at this detection point, amounting to almost 30% less in Trial B, provides clear evidence of the beneficial impact of the bio-protective strain shortly after its addition. Both trials exhibited a comparable trend in dissolved oxygen during clarification; however, the disparity between the treatments persisted until the start of the clarification process, after which the differences leveled out. Furthermore, the dissolved oxygen concentrations exhibited a further decline at the inoculum stage for both trials. In particular, Trial A showed a reduction of 19.49% from the previous detection point, with a value of 1.02 mg/L. Trial B exhibited an even more pronounced reduction, with a decrease of 30.33% from the previous detection point, thus resulting in a value of 0.74 mg/L of dissolved oxygen at the inoculum stage. Additionally, slight differences between the treatments were evident at this detection point and at the end of alcoholic fermentation, as highlighted by the ANOVA.

**Figure 5.**
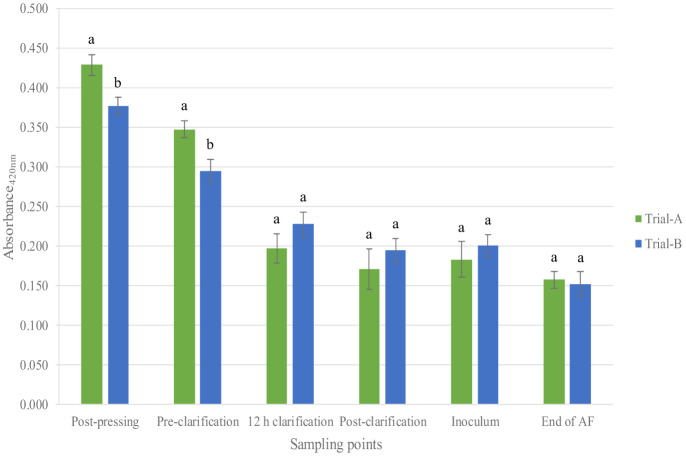
Bar chart of absorbance detected in must during pre-fermentative phase. Result indicates mean value ± standard deviation of two determinations from three replicates. Data within a sampling point followed by the same letter are not signiﬁcantly different according to Tukey's test. Data followed by different letters are significantly different (P value < 0.05). Abbreviations: AF, alcoholic fermentation; n.s., not significant.

In Figure 5, the absorbance values at 420 nm can be observed. The absorbance consistently decreased as the clarification proceeded. Following the pressing stage, Trial A showed significantly higher values (0.429) than Trial B (0.378). These findings slightly exceed the range reported by Darias-Martín et al. [44], who observed values between 0.2 to 0.6 after pressing, albeit with the addition of 40 mg/L of potassium metabisulphite immediately post-pressing. The trend persisted at the second detection point, where Trail B demonstrated the bioprotective effect of the HD A54 strain, thus yielding a lower value (0.295) than Trial A (0.348).

Clarification, a common winemaking procedure, aims to enhance the wine appearance by reducing browning issues and achieving a greater clarity [45]. Interestingly, no significant differences were found between the treatments from this point until the end of alcoholic fermentation. Both the dissolved oxygen and absorbance analyses underscore the positive impact of the *S. cerevisiae* HD A54 strain in protecting against oxidative processes in the musts, especially during the phase between pressing and clarification.

### 3.3. VOCs

Table 2 reports the VOC composition, which consists of 30 compounds grouped into six classes: Alcohols, aldehydes, ketones, carboxylic acids, esters, and others. Alcohols were the most abundant, with Trial A containing a total of 117.58 ± 12.78 mg/L and Trial B containing a total of 189.49 ± 12.63 mg/L. Among the alcohol compounds, hydroxyethylbenzene exceeded the perception threshold. This compound is linked to floral odors [46],[47]. Notably, Trial B had a higher concentration of this compound (135.31 mg/L) compared to Trial A (44.23 mg/L). Additionally, 3-methyl-thio-1-propanol was more pronounced in Trial A (2.01 mg/L) than in Trial B, where it remained below the perception threshold. This particular compound is associated with an unpleasant odor reminiscent of a cooked potato [48]. The differences in the VOC composition between the two trials suggest that the HD A54 strain may have positively influenced Trial B.

**Table 2.** Volatile organic compounds of experimental wines.

LRI	Compounds (Common name)	Odour Perception Threshold	Trial A (mg/L)	Trial B (mg/L)	
					S.S.
	**∑Alcohols**		**117.58 ± 12.78b**	**189.49 ± 12.63a**	*
607	2-methyl-1-propanol	40 mg/L [49]	1.00 ± 0.41a	0.00 ± 0.00b	*
764	1-pentanol	80 mg/L [50]	59.92 ± 5.32a	50.13 ± 4.08a	n.s.
765	1,2-propanediol	Unknown	0.52 ± 0.07a	0.86 ± 0.14a	n.s.
824	2,3-butanediol	120 mg/L [51]	2.88 ± 0.27a	0.00 ± 0.00b	***
856	3-methyl-1-pentanol	Unknown	0.25 ± 0.08a	0.14 ± 0.03a	n.s.
878	1-hexanol	8 mg/L [52]	0.91 ± 0.08a	0.45 ± 0.09b	*
983	3-methyl-thio-1-propanol	1 mg/L [53]	2.01 ± 0.09a	0.85 ± 0.04b	***
1134	Hydroxyethylbenzene	10 mg/L [46],[47]	44.23 ± 5.29b	135.31 ± 7.97a	***
1314	2-methoxy-4-methyl-phenol	Unknown	0.82 ± 0.05a	0.36 ± 0.11b	***
1432	4-hydroxyphenyl ethanol	Unknown	4.11 ± 1.05a	0.91 ± 0.09b	*
1505	2,4-di-tert-butyl phenol	0.2 mg/L [50]	0.93 ± 0.07a	0.48 ± 0.08b	*
	**∑Aldehydes**		**3.03 ± 0.44a**	**2.16 ± 0.40a**	**n.s.**
1224	3,4-dimethyl benzaldehyde	Unknown	0.80 ± 0.15a	0.51 ± 0.12a	n.s.
2020	Octadecanal	Unknown	2.23 ± 0.29a	1.65 ± 0.28a	n.s.
	**∑Ketones**		**2.04 ± 0.54a**	**1.56 ± 0.30a**	**n.s.**
722	3-hydroxy-2-butanone	30 mg/L [49]	0.49 ± 0.00a	0.51 ± 0.12a	n.s.
964	4-hydroxy-2-butanone	Unknown	1.03 ± 0.51a	0.74 ± 0.11a	n.s.
1285	2-hydroxy-2-methyl-1-phenyl-1-propanone	Unknown	0.52 ± 0.03a	0.31 ± 0.07b	*
	**∑Carboxylic acids**		**4.47 ± 0.53a**	**0.94 ± 0.16b**	**
590	Acetic acid	200 mg/L [54]	0.63 ± 0.08a	0.00 ± 0.00b	*
916	4-hydroxy butanoic acid	Unknown	0.36 ± 0.06a	0.12 ± 0.04b	**
1015	Hexanoic acid	0.42 mg/L [52]	0.27 ± 0.08a	0.00 ± 0.00b	**
1195	Octanoic acid	2.20 mg/L [55]	3.21 ± 0.31a	0.82 ± 0.12b	**
	**∑Esters**		**15.85 ± 3.50a**	**15.82 ± 2.39a**	**n.s.**
589	Ethyl acetate	7.5 mg/L [54]	8.25 ± 0.99a	2.14 ± 0.62b	**
803	Ethyl lactate	60 mg/L [56]	0.15 ± 0.02a	0.29 ± 0.06a	n.s.
886	3-methyl-butyl acetate	0.03 mg/L [52]	2.99 ± 0.86b	7.84 ± 0.53a	***
941	Ethyl 3-hydroxy butanoate	Unknown	0.21 ± 0.08a	0.16 ± 0.04a	n.s.
1002	Ethyl-butyl acetate	Unknown	1.40 ± 0.62a	0.76 ± 0.06a	n.s.
1199	Ethyl octanoate	0.005 mg/L [52]	0.66 ± 0.21b	1.40 ± 0.23a	**
1260	2-phenylethyl acetate	0.25 mg/L [57]	1.51 ± 0.63b	3.08 ± 0.77a	*
1386	Ethyl-9-decenoate	Unknown	0.61 ± 0.07a	0.15 ± 0.08b	*
1395	Ethyl decanoate	0.2 mg/L [52]	0.07 ± 0.02a	0.00 ± 0.00b	*
	**∑Others**		**0.25 ± 0.05a**	**0.17 ± 0.07a**	**n.s.**
1768	3-(2-hydroxyethyl)-indole	Unknown	0.25 ± 0.05a	0.17 ± 0.07a	n.s.

Result indicates mean value ± standard deviation of two determinations from three replicates. Data within a row followed by the same letter are not signiﬁcantly different according to Tukey's test. P value: *, P < 0.05 **, P < 0.01; ***, P < 0.001. Abbreviations: SS, statistical significance; n.s., not significant.

As the second most prevalent class of compounds, the esters had concentrations of 15.85 ± 3.50 for Trial A and 15.82 ± 2.39 mg/L for Trial B. Ethyl acetate, a prominent ester, exhibited significantly higher levels in Trial A (8.25 mg/L) compared to Trial B (2.14 mg/L), which remained below the threshold [54]. Interestingly, ethyl acetate serves as an indicator of oxidation: lower ester values correlate with a greater protection against oxidation [58]. In terms of fruity aromas, Trial B stood out due to the presence of 3-methyl-butyl acetate (banana-associated) [59] and ethyl octanoate (with pear and pineapple notes) [59], measuring 7.84 mg/L and 1.40 mg/L, respectively. Although Trial A recorded values above the perception threshold, they were significantly lower than those in Trial B (2.99 and 0.66 mg/L, respectively). Additionally, Trial B exhibited a higher concentration of 2-Phenylethyl acetate, an ester associated with flowery notes [47], at 3.08 mg/L, compared to Trial A at 1.51 mg/L.

Another represented class was carboxylic acids, which are often linked to unwanted odors in wine [30]. Surprisingly, all carboxylic acids remained below the perception threshold in this study. However, it is essential to note that hexanoic acid and octanoic acid are typically responsible for unpleasant odors such as fatty, cheesy, and rancid smells. In the bio-protected treatment (Trial B), these acids were either entirely absent or below the perception threshold. In Trial A, hexanoic acid remained below the threshold (0.42 mg/L), while octanoic acid was perceptible at a concentration of 3.21 mg/L. Furthermore, acetic acid, a key indicator of wine spoilage and quality reduction [60], remained below the perception threshold in Trial A and was totally absent in Trial B.

### 3.4. Sensory analysis

**Figure 6.**
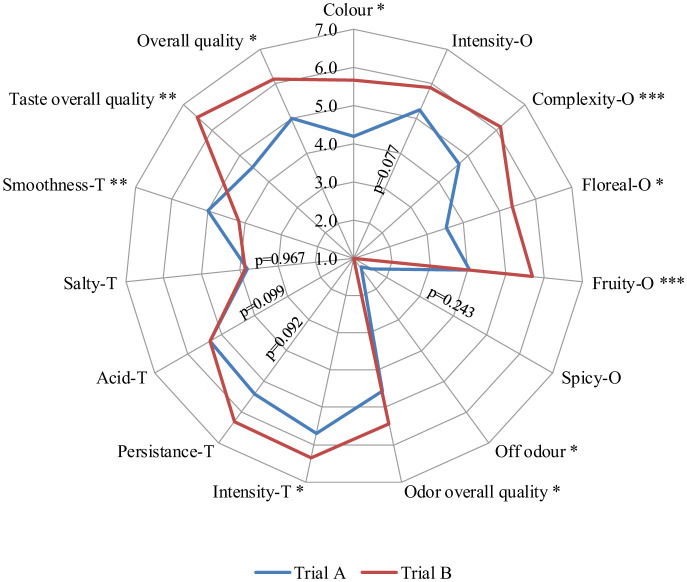
Radar plot generated by sensory analysis conducted on experimental wines. Result indicates mean value ± standard deviation of two determinations from three replicates. P value: *, P < 0.05 **, P < 0.01; ***, P < 0.001. Abbreviations: T, taste; O, odour.

Figure 6 displays a radar plot generated after a sensory evaluation of the experimental wines. Notably, the two treatments showed variability, particularly in the odor attributes. Bio-protected Trial B scored higher in several odor-related attributes, except for the “off-odor” attribute, where Trial A had a slightly greater score. The “fruity” attribute received a definitively higher score for Trial A, thus aligning with the VOC analysis that highlighted compounds associated with fruity aromas, such as 3-methyl-butyl acetate and ethyl octanoate. Similarly, in terms of the floral attribute, the judges favored Trial B, which is consistent with the VOC analysis that showed higher values for hydroxyethylbenzene and 2-phenylethyl acetate, both associated with floral scent, specifically rose.

Considering these findings, Trial B scored better in terms of complexity and the overall odor quality. Interestingly, the scores for fruity and floral attributes closely resembled those reported in a study by Alfonzo et al. [41] on Catarratto wines produced with different *S. cerevisiae* strains. As for the taste descriptors, Trial B received a higher score for intensity, while Trial A scored better in the smoothness attribute. Although mouthfeel-related attributes are often linked to a higher concentration of glycerol produced by certain non-*Saccharomyces* yeast strains [61],[62], this study did not highlight significantly higher glycerol production in Trial A nor a greater non-*Saccharomyces* yeast population. Nevertheless, Trial B received a higher score for the descriptor “taste overall quality”. Overall, the judges preferred the attributes associated with Trial B.

## 4. Conclusions

In recent years, reducing SO_2_ levels in winemaking has gained importance. Bioprotective yeasts offer winemakers a valuable solution by limiting the growth of unwanted microorganisms, preserving the product quality, and optimizing the space utilization. Current research primarily focuses on non-*Saccharomyces* yeasts such as *Torulaspora delbrueckii* and *Metschnikowia pulcherrima* to play a bio-protection role and to enhance the sensory aspects, which are particularly crucial in this context. The use of non-*Saccharomyces* yeasts could deeply influence the wine quality, thereby producing peculiar metabolites and potentially affecting other wine parameters such as acidity and alcohol content. This study evaluated the bio-protective properties of a specific *S. cerevisiae* strain which was added during the pre-fermentative stage throughout winemaking. Specifically, the addition of the HD A54 *S. cerevisiae* strain positively influenced the dissolved O_2_ levels from post-pressing until the end of fermentation. Additionally, it impacted the absorbance values, particularly before clarification, thus mitigating the browning effects. Furthermore, the VOC composition revealed an enhanced protection in the bio-protected trial, specifically for compounds associated with fruity and floral aromas. A sensory evaluation by judges confirmed these findings, with Trial B receiving higher scores for floral and fruity attributes. Overall, the wines treated with *S. cerevisiae* HD A54 after pressing were favorably evaluated. Finally, this strain successfully facilitated must fermentation without the need for SO_2_, thus suggesting its potential use in wine fermentation to either eliminate or decrease the utilization of sulphites.

## Use of AI tools declaration

The authors declare they have not used Artificial Intelligence (AI) tools in the creation of this article.
